# A Proposed Concept for Defective Mitophagy Leading to Late Stage Ineffective Erythropoiesis in Pyruvate Kinase Deficiency

**DOI:** 10.3389/fphys.2020.609103

**Published:** 2021-01-20

**Authors:** Annelies Johanna van Vuren, Eduard Johannes van Beers, Richard van Wijk

**Affiliations:** ^1^Van Creveldkliniek, Division of Internal Medicine and Dermatology, University Medical Center Utrecht, Utrecht University, Utrecht, Netherlands; ^2^Central Diagnostic Laboratory-Research, University Medical Center Utrecht, Utrecht University, Utrecht, Netherlands

**Keywords:** reticulocytes, pyruvate kinase deficiency, ferroptosis, mitophagy, GPX4

## Abstract

Pyruvate kinase deficiency (PKD) is a rare congenital hemolytic anemia caused by mutations in the *PKLR* gene. Here, we review pathophysiological aspects of PKD, focusing on the interplay between pyruvate kinase (PK)-activity and reticulocyte maturation in the light of ferroptosis, an iron-dependent process of regulated cell death, and in particular its key player glutathione peroxidase 4 (GPX4). GPX4 plays an important role in mitophagy, the key step of peripheral reticulocyte maturation and GPX4 deficiency in reticulocytes results in a failure to fully mature. Mitophagy depends on lipid oxidation, which is under physiological conditions controlled by GPX4. Lack of GPX4 leads to uncontrolled auto-oxidation, which will disrupt autophagosome maturation and thereby perturb mitophagy. Based on our review, we propose a model for disturbed red cell maturation in PKD. A relative GPX4 deficiency occurs due to glutathione (GSH) depletion, as cytosolic L-glutamine is preferentially used in the form of *α*-ketoglutarate as fuel for the tricarboxylic acid (TCA) cycle at the expense of GSH production. The relative GPX4 deficiency will perturb mitophagy and, subsequently, results in failure of reticulocyte maturation, which can be defined as late stage ineffective erythropoiesis. Our hypothesis provides a starting point for future research into new therapeutic possibilities, which have the ability to correct the oxidative imbalance due to lack of GPX4.

## Pyruvate Kinase Deficiency

### Pyruvate Kinase Deficiency: A Rare Hereditary Hemolytic Anemia

Pyruvate kinase deficiency (PKD) is a rare form of hereditary hemolytic anemia. It is the most common glycolytic enzymopathy and thereby an important cause of hereditary non-spherocytic hemolytic anemia (Valentine et al., 1961; Germain, 1962; Tanaka et al., 1962; Brunetti et al., 1963; Oski and Diamond, 1963; Grace et al., 2019a), with an estimated prevalence of clinically diagnosed PKD patients between 3.2 and 8.5 per million in Western populations (Secrest et al., 2020).

The disease is caused by compound heterozygous or homozygous loss of function mutations in the *PKLR* gene encoding for liver and red blood cell specific pyruvate kinase (PK-R; Canu et al., 2016; Grace et al., 2019a). Over the last decades more than 300 different pathogenic variants have been reported (Bianchi and Fermo, 2020). In the last step of glycolysis, PK converts phosphoenolpyruvate (PEP) into pyruvate, thereby generating ATP, the sole source of energy of the mature red blood cell. Consistent with the concomitant decrease in PK-R activity as a result of mutation, PKD leads to a loss of ATP and, retrograde accumulation of glycolytic intermediates (Rab et al., 2020).

Under physiological conditions, both PK-R and PK-M2 are expressed in basophilic erythroblasts. The latter isozyme is produced from another gene, *PKM*. During further erythroid differentiation and maturation, a switch in isoenzymes occurs whereby progressively increased PK-R expression gradually replaces PK-M2 (Gray et al., 2014). Pyruvate in reticulocytes is ultimately destined for transport into mitochondria as master fuel for the tricarboxylic acid (TCA) cycle carbon flux. In reticulocytes, pyruvate can also be derived from additional sources in cellular cytoplasm (e.g., oxidation of lactate and transamination of alanine). Pyruvate enters the TCA cycle as citrate or oxaloacetate. Modulation of pyruvate entrance balances anaplerotic carbon entrance and cataplerotic carbon exit to ensure continuous cycle of the TCA cycle carbon flux. Pyruvate drives ATP production in the mitochondria by oxidative phosphorylation and other pathways intersecting the TCA cycle (Gray et al., 2014). Mature erythrocytes lack nuclei and mitochondria, and are therefore incapable to generate energy *via* the TCA cycle. Consequently, mature erythrocytes fully depend on anaerobic conversion of glucose by the Embden-Meyerhof pathway for generation of ATP (van Wijk and van Solinge, 2005).

Pyruvate kinase deficiency is characterized by molecular as well as clinical heterogeneity. Clinical features of PKD vary widely and range from mild anemia to red cell transfusion dependency. The relationship between genotype and phenotype is still incompletely understood (Bianchi and Fermo, 2020; Bianchi et al., 2020). The presence of compound heterozygous pathogenic missense mutations may lead to the presence of several different combinations of PK tetramers each with its own kinetic, allosteric, and structural properties (van Wijk et al., 2009). Thereby, individual differences in metabolic or proteolytic activity, differences in splenic function, or variation in expression of (compensating) PK isoenzymes and the activity of other compensating pathways may contribute to the disease phenotype, as well as epigenetic factors and co-inheritance of other (red cell) diseases (Bianchi and Fermo, 2020). Treatment for PKD is generally supportive including splenectomy, blood transfusion, and iron chelation as main therapies. However, new therapies are under investigation, for example, mitapivat, a small-molecule allosteric activator of PK has shown to induce a clinically relevant hemoglobin increase and improvement in hemolytic parameters in a phase II clinical trial (Grace et al., 2019b).

Remarkably, in PKD post-splenectomy reticulocyte counts tend to be extremely high, varying with hemoglobin response. Most of the patients (78%) were able to discontinue regular transfusions after splenectomy, and in this group, the median post-splenectomy reticulocyte count was 32.3% (range 4.8–65%). This brisk reticulocytosis is likely due to prolonged reticulocyte survival. Patients still requiring regular red cell transfusions after splenectomy tended to have lower reticulocyte counts post-splenectomy (20.9%, range 2.5–44.1%; Nathan et al., 1968; Grace et al., 2018). Notably, the high reticulocyte count post-splenectomy contrasts with the decrease in reticulocytes after splenectomy in other hemolytic anemias, such as hereditary spherocytosis (Leblond et al., 1978a,b).

### Reticulocyte Features in PKD: Historic Observations

Radioisotope studies on PKD patients showed that red blood cells are heterogeneously affected, with a more seriously damaged population of younger cells, which sequestrate in the spleen and are subsequently destructed by the reticuloendothelial system. Splenectomy improved survival of these younger cells, although destruction still took place on a slower rate in the liver. More mature PK-deficient red blood cells were able to survive longer (Nathan et al., 1968; Mentzer et al., 1971).

In PKD patients, splenectomy resulted in considerable changes in red cell morphology. In sharp contrast with the paucity of red cell abnormalities before splenectomy, post-splenectomy samples from peripheral blood showed ecchinocytes, immature reticulocytes (with remaining organelles), and thin macrocytic discocytes (Leblond et al., 1978a,b).

In another study on 17 PKD patients, mutant PK was more susceptible to inhibition by ATP. Consumption of glucose and formation of lactate were lower in PK-deficient red blood cells when compared to PK-sufficient red cell populations with similar reticulocyte counts. In post-splenectomized PK-deficient patients with higher degrees of reticulocytosis more severe impairment of glucose metabolism was observed (Schroter et al., 1977).

These reports further underline selective destruction of relatively young PK-deficient reticulocytes by the spleen. The mechanism(s) involved in this unique pathophysiological feature of PKD is not understood. In an attempt to provide a rationale, we focus here on the interplay between PK-activity and reticulocyte maturation in the light of ferroptosis, an iron-dependent process of regulated cell death, and in particular, its key player glutathione peroxidase 4 (GPX4). Based on this, we propose a model for disturbed red cell maturation in PKD that suggests that the main problem in PKD may not only be the PK-deficient erythrocyte, but for it to reach the mature red cell state.

## Glutathione Peroxidase 4

### Red Cells and Oxidative Stress

Oxidative stress is defined as an imbalance in oxidants and reductants in favor of the oxidants. The presence of high concentrations of oxygen and iron in red blood cells exposes them constantly to oxidative stress as reactive oxygen species (ROS) are formed. Both heme and iron catalyze the Fenton reaction that produces the highly reactive hydroxyl radical from the interaction between superoxide and hydrogen peroxides. Excessive ROS production leads to serious damage to organelles and DNA, or induces programmed cell death (e.g., ferroptosis). To provide protection against ROS, repair oxidative damage and maintain a reducing intracellular environment, (developing) red cells are equipped with a variety of antioxidants, including superoxide dismutase 1 and 2, catalase, glutathione peroxidase 1, peroxiredoxin I and II, and glutathione (GSH)-synthesizing enzymes (van Zwieten et al., 2014; Canli et al., 2016).

**Figure 1:**
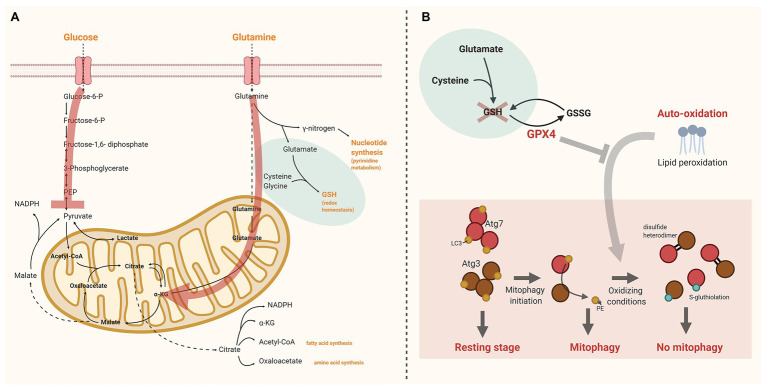
The interplay between glutathione peroxidase 4 (GPX4), pyruvate kinase deficiency (PKD), and reticulocyte maturation. **(A)** Schematic overview of glucose and glutamine uptake and metabolism in reticulocytes. The red arrows indicate the alterations in PKD. Pyruvate kinase (PK) facilitates the final step of glycolysis converting phosphoenolpyruvate (PEP) into pyruvate. In PKD anaplerotic carbon entrance *via* other routes than pyruvate is essential to continue tricarboxylic acid (TCA) cycling. Therefore, cytosolic glutamine is preferentially used to form *α*-ketoglutarate (α-KG) at the expense of glutathione (GSH) production. **(B)** Under physiologic conditions lipid peroxidation in reticulocytes is controlled by GPX4. GPX4 depletion may lead to irreversible oxidation of Atg3 and Atg7. This impedes the LC3 lipidation process, essential for autophagosome formation and maturation. The red cross illustrates the presumed lack of GSH in PKD. As a consequence GPX4 function is blocked. Created with BioRender.com. α-KG, α-ketoglutarate; GPX4, glutathione peroxidase 4; GSH, glutathione; GSSG, glutathione disulfide; PEP, phosphoenolpyruvate; and PKD, pyruvate kinase deficiency.

Glutamine is an important metabolic fuel for rapidly proliferating cells to meet the cell’s demand for ATP, biosynthetic precursors, and reducing agents. When transferred to the mitochondrion, glutamine is converted to glutamate to serve as fuel for the TCA cycle when converted to *α*-ketoglutarate (anaplerotic carbon entrance). During hypoxia α-ketoglutarate may also be converted to citrate, which is used for fatty acid synthesis, amino acids synthesis, or production of the reducing agent NADPH. In the cytosol, glutamine can also be converted to glutamate by donation of its *γ*-nitrogen for nucleotide synthesis (pyrimidine metabolism). Cytosolic glutamate is critical for GSH synthesis, which in turn functions as reducing substrate for GPX4, an essential enzyme to protect the cell against oxidative damage (Jin et al., 2016; Xiao et al., 2016; Figure 1A).

12/15-Lipoxygenases in mice, and its human homolog 15-lipoxygenase, and GPX4 act antagonistically. Lipoxygenases generate highly reactive peroxidation products of unsaturated fatty acids prone to further lipid peroxidation and GPX4 reduces lipid peroxidation products in membranes to stable hydroxyl-derivatives (Kuhn and Borchert, 2002). GPX4 plays an important role in red cell development, as two recently published papers showed that *Gpx4*-knockout mice are phenotypically characterized by ineffective erythropoiesis (Canli et al., 2016; Altamura et al., 2020), and disrupted reticulocyte maturation (Altamura et al., 2020).

### The Role of GSH Depletion and GPX4 in Ferroptosis

Ferroptosis has recently emerged as a new cell death modality. The importance of this newly recognized process for a variety of diseases, including neuron degenerative diseases, cancer, and ischemic organ damage, has been shown. Ferroptosis is triggered by iron-dependent lipid peroxidation, in reactions catalyzed by iron and ROS. GPX4, and its cofactor GSH, function in the defense against ferroptosis by functioning as reducing agents required for clearance of lipid ROS. GSH requires cysteine for its synthesis from glutamine. Cellular import of cysteine is coupled to the export of glutamate *via* System X_C_^−^ (SLC7A11; cysteine/glutamate antiporter). Upon blocking this system by the synthetic small-molecule erastin the import of cysteine is prevented. The resulting cysteine deprivation impairs synthesis of GSH, and thereby causes increased glutaminolysis. Glutaminolysis is a major source of anaplerosis; *α*-ketogluterate is the TCA cycle metabolite immediately downstream glutaminolysis. Mitochondrial TCA cycle activity (and subsequent mitochondrial hyperpolarization), and action of the electron transport chain are required for potent ferroptosis (Dixon et al., 2012; Gao et al., 2019; Bock and Tait, 2020). Oxidative phosphorylation requires a coordinated transfer of electrons through the complexes of the electron transport chain thereby generating a proton gradient across the inner mitochondrial membrane. Excessive promotion of glutaminolysis (e.g., by cysteine deprivation) stimulates TCA cycle activity and therefore potently enhances mitochondrial respiration leading to mitochondrial hyperpolarization and increased production of ROS, which ultimately promotes lipid peroxidation and, hence, accelerates ferroptosis. This accounts particularly for developing red cells: proximity of mitochondrial membranes to sources of free iron (heme-containing proteins) and ROS makes them important targets for lipid oxidation (Dixon et al., 2012; Gao et al., 2019; Bock and Tait, 2020).

Metabolic profiling has shown that GSH depletion is one of the mechanisms by which ferroptosis is initiated. Inactivation of GPX4, through depletion of GSH or by using GPX4 inhibitors, ultimately results in overwhelming lipid peroxidation and cell death in various non-erythroid cells (Yang et al., 2014; Stockwell et al., 2017). N-acetylcysteine (NAC), a biosynthetic precursor to GSH, in turn has shown to prevent cell death induced by GSH-inhibiting agents (Yang et al., 2014).

Several trials have been conducted with NAC that specifically focused on congenital red cell diseases. *In vitro* NAC amide, the amide form of NAC with improved membrane permeability, possessed the capacity to replenish GSH in red cells from ß-thalassemic patients and reduce the amount of ROS (Amer et al., 2008). This reducing capacity of NAC was confirmed *in vivo* in children diagnosed with ß-thalassemia major: parameters of oxidative stress declined and erythrocyte life span improved (Ozdemir et al., 2014). In sickle cell disease (SCD), NAC supplementation significantly lowered erythrocyte phosphatidylserine (PS) expression, measured as representative of erythrocyte membrane (oxidative) damage (Nur et al., 2012). In red cells, extensive lipid peroxidative damage to the red cell inner membranes will lead to abnormal PS externalization (Banerjee and Kuypers, 2004; Hannemann et al., 2018; Al Balushi et al., 2019). These, and several other studies on NAC, underlined the importance of maintaining cellular redox balance for red cell survival as imbalanced ROS production may lead to oxidative stress and (premature) cell death (Wright et al., 1998; Amen et al., 2017).

In an attempt to reduce excessive oxidative stress in sickle red cells another compound gained interest: L-glutamine. Glutamine is a precursor for synthesis of GSH, nicotinamide adenine dinucleotide (NAD), and arginine, which all protect red cells against oxidative damage and (indirectly) maintain vascular tone (Quinn, 2018). Sickle red cells harbor a decreased NAD redox potential (Zerez et al., 1988; Niihara et al., 1997). GSH consumption is increased in response to oxidative stress without evidence for substrate-limited synthesis of GSH (Kiessling et al., 2000; Morris et al., 2008). In the presence of sufficient amounts of substrate, the rationale for L-glutamine supplementation to replenish GSH is limited. However, pharmaceutical doses of L-glutamine did increase the NAD redox potential in sickle red cells (Niihara et al., 1998). After several smaller trials, as summarized by others (Quinn, 2018), a phase 3 clinical trial led in 2017 to the approval of L-glutamine for patients with SCD in order to reduce sickle cell-related acute pain crises and hospitalizations (Niihara et al., 2018). Whether the efficacy of L-glutamine is related to modulation of GSH metabolism remains disputable, and various other unanswered questions remain regarding (long-term) efficacy and safety (including effects on non-erythroid cells), as well as its acceptance by SCD patients (Quinn, 2018).

Altogether, both the studies with NAC and L-glutamine in red cell disorders do underscore the importance of appropriate redox regulation in the (maturing) red cells which, in the presence of heme and iron, is continuously subjected to auto-oxidation, including lipid peroxidation.

## Reticulocyte Maturation

### The Role of Mitophagy in Reticulocyte Maturation Under Physiological Conditions

Enucleated reticulocytes exit the bone marrow, circulate in the blood for 2–3 days and then mature mostly in the spleen into mature erythrocytes. Throughout erythroid maturation mitochondria are progressively lost. The mechanism involved is a specialized form of macro-autophagy, called mitophagy: a selective process by which damaged and depolarized mitochondria are removed and degraded (Mortensen et al., 2010a). In mammalian reticulocytes mitophagy occurs in response to developmentally programmed changes in the cell (Mortensen et al., 2010a,b; Grosso et al., 2017). In short, mitochondrial membrane depolarization in response to the mitochondrial protein BNIP3L (BCL2 interacting protein 3 like; NIX; Zhang et al., 2012), upregulated during terminal red cell differentiation, signals activation of canonical autophagy proteins (Atg). This initiates the formation of *de novo* double membranes, termed phagophores. Phagophores assemble around depolarized mitochondria which, upon closure, form the autophagosome. Finally, the autophagosome fuses with lysosomes to degrade their content or fuses with the plasma membrane for exocytosis. Several Atg proteins are involved in the assembly of the phagophore. Inhibition of mammalian target of rapamycin (mTOR) is at the start of autophagosome formation, as it activates unc-51 like autophagy activating kinase 1 (ULK1), facilitator of the Atg5/Atg7 pathway, and releases beclin1 (Frudd et al., 2018). Growth and maturation of the autophagosome is mediated by the Atg5–Atg12 pathway and by the microtubule-associated protein 1 light chain 3 (LC3, the mammalian homolog of yeast Atg8) pathway, both of which depend on Atg7 (Honda et al., 2014). When autophagy is inactive, Atg3 and Atg7 form a stable thioester with LC3. Maturation and growth of the autophagosome requires that phosphatidylethoanolamine (PE) is conjugated to LC3, a process called lipidation, this will occur in response to autophagic stimuli sensed by Atg3. In response to autophagic stimuli, the stable thioesters between LC3 and the catalytic thiols on both Atg3 and Atg7 become transient, which promotes LC3 lipidation (Burgoyne, 2018; Frudd et al., 2018). The lipidated form of LC3 anchors to the phagophore membrane. Recognition of target mitochondria by the autophagosome occurs through LC3. BNIP3L on mitochondrion targeted for mitophagy has two lipidated LC3 binding sites, and thereby plays an important role in total engulfment of the mitochondrion and completion of autophagosome formation (Novak et al., 2010; Moras et al., 2017). Besides this so-called Atg5/Atg7 pathway, several alternative pathways for initiation of the mitophagy process have been proposed with varying contributions to total mitophagy: 15-lipoxygenase is one of the central enzymes in an alternative pathway. 15-lipoxygenase is highly expressed in reticulocytes. However, its contribution to mitochondrial clearance seems to be only modest when compared to the classical Atg5/Atg7-dependent pathway as 12/15-lipoxygenase knockout mice have normal erythrocyte and reticulocyte counts (van Leyen et al., 1998; Grullich et al., 2001; Mortensen et al., 2010a).

### GPX4 Deficiency Results in Ineffective Erythropoiesis and Reticulocytosis

Canli et al. (2016) showed that loss of GPX4 causes erythroid precursor cell death and anemia in mice, due to ineffective erythropoiesis. Surprisingly, erythroid precursor cell death was triggered *via* RIP3-dependent necroptosis, and not *via* ferroptosis. Increased lipid peroxidation and oxidative stress in erythroid *Gpx4*-knockout cells did not impair their lifespan in the peripheral blood stage. However, it did increase the number of reticulocytes, suggesting defective maturation of reticulocytes. In contrast to these findings, GPX4 inhibition did not impact cell death during human erythroblast differentiation, as recently published by Ouled-Haddou et al. (2020). This may suggest that human erythroblasts rely on other enzymes with GPX-activity that are able to overcome the effects of GPX4 inhibition. Notably, GPX4 inhibition in human erythroblasts did impair enucleation in a ferroptosis-independent, necroptosis-independent, and mitophagy-independent manner by disrupting lipid raft clustering and myosin-regulatory light-chain phosphorylation required for contractile ring assembly and cytokinesis. Unfortunately, the influence of GPX4 on the reticulocyte stage of red cell development was not investigated.

In a mice model with exclusive knockout of *Gpx4* in the hematopoietic system, Altamura et al. (2020) confirmed that lack of GPX4 resulted in a phenotype of ineffective erythropoiesis with increased numbers of immature reticulocytes in line with Canli et al. (2016). The failure to proceed toward maturation was associated with increased lipid peroxidation (remnants of) mitochondria were seen in GPX4 deficient reticulocytes. These studies were the first to show the link between GPX4 and impaired reticulocyte maturation, of which the following three key elements can be distinguished:

1. Lipid peroxidation. Lack of 12/15-lipoxygenase (or its human homolog 15-lipoxygenase) was not sufficient to disrupt reticulocyte maturation (Sun and Funk, 1996). However, 12/15-lipoxygenase was shown to be involved in autophagy processes in murine macrophages (Morgan et al., 2015). In reticulocytes, taking into account that red cells carry high concentrations of iron and heme, highly expressed lipoxygenases may contribute to the initiation of lipid hydroxyperoxide production, but mainly sensitize the cell for iron-catalyzed spontaneous (non-enzymatic) peroxyl radical-mediated chain reactions called auto-oxidation (Shah et al., 2018; Altamura et al., 2020).
2. GPX4. Under physiologic conditions lipid peroxidation in reticulocytes is controlled by GPX4. Loss of GPX4 leads to uncontrolled lipid peroxidation with the highest levels of lipid peroxidation in immature reticulocytes when both GPX4 and vitamin E (known to antagonize peroxide production) were deficient (Canli et al., 2016; Altamura et al., 2020).
3. LC3 lipidation. Loss of GPX4 may lead to irreversible oxidation of Atg3 and Atg7. The loss of a stable covalent interaction of Atg3/Atg7, and LC3 in response to autophagy induction makes their catalytic thiols more susceptible for oxidation. If catalytic thiols are available on both Atg3 and Atg7, oxidation will result in formation of a disulfide heterodimer; oxidation of a single catalytic thiol is more likely to result in formation of a stable S-gluthiolation. The irreversible oxidation of catalytic thiols on Atg3 and Atg7 prevents the lipidation process of LC3, and thereby inhibits its function in autophagosome maturation (Burgoyne, 2018; Frudd et al., 2018; Figure 1B).

Ultimately, this sequence of events will lead to severely perturbated mitophagy and, therefore, a defect in reticulocyte maturation.

## Hypothesis: PKD Leads to Defective Reticulocyte Maturation

Based on the above, we hypothesize that in PKD the young reticulocyte is fully dependent on its reduced PK-R activity to provide the cell with pyruvate to fuel the TCA cycle. In PKD, anaplerotic carbon entrance *via* other routes than pyruvate is essential. Glutaminolysis will be the major source of anaplerotic carbon entrance in the absence of sufficient amounts of pyruvate. As cytosolic glutamine is preferentially used in the form of *α*-ketogluterate to fuel the TCA cycle, there will be shortage of glutamine for synthesis of GSH and, subsequently, bioavailability of GPX4 will be impaired. The red cells are, in the presence of heme and iron, continuously subjected to auto-oxidation, including lipid peroxidation. In developing red cells mitophagy pathways are triggered to fulfill the maturation process. Excessive auto-oxidation in the absence of GPX4 impairs the LC3 lipidation process when autophagy is stimulated by rendering Atg3 and Atg7 inactive as a consequence of catalytic thiol oxidation. Ultimately, this process will perturbate mitophagy, and subsequently halts the reticulocyte maturation process, as was observed in models of *Gpx4* loss (Canli et al., 2016; Altamura et al., 2020; Figure 1).

In line with the observations of Ouled-Haddou et al. (2020) described above a shortage of GPX4 bioavailability in erythroblasts may impair enucleation, which fits with the presence of nucleated red cells in the peripheral blood of PKD patients. However, persistent expression of PK-M2, a suggested phenotypic modifier (Grace et al., 2015) may limit disrupted enucleation in PK-R-deficient erythroid cells as a relevant reduction in GSH bioavailability in the presence of PK-M2 is not expected.

Depending on the underlying mutation and other (non-)genetic factors, as discussed in section I, *PKD: a rare hereditary hemolytic anemia*, residual activity of PK will vary among patients, and even between individual cells in one patient. This is anticipated to lead to varying degrees of glutaminolysis required for anaplerosis, and a variable reduction of GSH synthesis and GPX4 bioavailability per cell. As a consequence, some reticulocytes will mature whereas in other reticulocytes the maturation process is blocked. Interestingly, earlier studies did not report an absolute decline in either GSH or GSSG in mature PK-deficient erythrocytes, but a decline in GSH/GSSG ratio was reported to result in an acquired reduction in PK-activity (van Berkel et al., 1973; Blume et al., 1974; Sprengers et al., 1977). Notably, glutaminolysis for anaplerotic carbon entrance is not required in mature red cells that are incapable of generating energy *via* the TCA cycle (van Wijk and van Solinge, 2005). In PKD, besides induction of a maturation defect, lack of GSH in PK-deficient reticulocytes may further disrupt the already reduced PK-activity by oxidation of the enzyme, thereby initiating a deleterious downward spiral (van Berkel et al., 1973; Blume et al., 1974; Sprengers et al., 1977).

As described in section I, *Reticulocyte features in PKD: historic observations*, earlier observations showed that (non-maturing) young reticulocytes in PKD are degraded by the red pulp macrophages of the spleen. The three most commonly recognized signals in the interaction between red pulp macrophages and red cells, band 3, PS and CD47-SIRP*α*, are known to be influenced by oxidative stress. Thus, distinct oxidative-stress induced modifications of these molecules on PK-deficient reticulocytes may facilitate their accelerated clearance by the spleen (Banerjee and Kuypers, 2004; Arese et al., 2005; Burger et al., 2012; Klei et al., 2017; Hannemann et al., 2018; Al Balushi et al., 2019). Splenectomy abrogates this process, resulting in a slower rate of removal of immature reticulocytes in the liver, thereby extending the longevity of non-maturing reticulocytes, which provides an explanation for the profound reticulocytosis that is observed post-splenectomy in PKD patients. Individual variation in the degree of reticulocytosis, however, may exist, depending (at least partly) on the individual hepatic capacity to substitute for splenic clearance of non-maturing reticulocytes (Grace et al., 2018).

In conclusion, we postulate that defective reticulocyte maturation is a new key pathophysiological aspect of PKD that can be classified as late stage ineffective erythropoiesis. Here, the proposed hypothesis provides an exciting new area of research on the pathophysiology of PKD and can offer new therapeutic possibilities aimed at correcting the oxidative imbalance due to lack of GSH. Interventions that enhance PK-activity, e.g., mitapivat, could (partially) correct reticulocyte maturation. Other potentially attractive therapeutic strategies that could target the disrupted redox balance underlying halted reticulocyte maturation are amino-acid supplementation with L-glutamine, vitamin E supplementation, and NAC.

Supplementation of the amino acid L-glutamine may correct for the increased fuel demand of the TCA cycle. Additional supply of L-glutamine may restore GSH production in PKD, as we expect GSH synthesis in PKD to be substrate-limited. Supplementation of vitamin E could be beneficial because vitamin E interposes in lipid membranes, and acts as antagonist of lipid peroxide production through its high affinity for unpaired electrons. Vitamin E synergizes with GPX4 in correcting the action of lipid hydroxyperoxides, and as a consequence may therefore limit perturbation of mitophagy in PKD. Repletion of vitamin E deficiency was shown to partly correct lipid peroxidation in the presence of GPX4 deficiency; the role of supra-normal vitamin E doses, however, remains to be explored (Altamura et al., 2020). NAC may be a third way to restore disrupted mitophagy. Its supplementation has previously shown to prevent cell death induced by GSH-inhibiting agents (Yang et al., 2014). Trials in *β*-thalassemia and SCD patients provided prove for its capacity to replenish the amount of GSH and, accordingly, reduce levels of oxidative stress (Amer et al., 2008; Nur et al., 2012; Ozdemir et al., 2014). Notably, catabolism of other amino acids may also correct for the lack of anaplerotic carbon entrance into the TCA cycle in PKD, or replenish the amount of glutamate. Both alanine aminotransferase (alanine catabolism) and aspartate aminotransferase (aspartate catabolism) may contribute to glutamate biosynthesis (Ellinger et al., 2011; Whillier et al., 2011). Thereby, glutamate may also be derived from histidine, proline, and arginine catabolism (Owen et al., 2002). Future studies are warranted to investigate our hypothesis on the existence of late stage ineffective erythropoiesis in PKD as well as targeting it for therapeutic purposes.

## Data Availability Statement

The original contributions presented in the study are included in the article/supplementary material, further inquiries can be directed to the corresponding author.

## Author Contributions

All authors listed have made a substantial, direct and intellectual contribution to the work, and approved it for publication.

### Conflict of Interest

The authors declare that the research was conducted in the absence of any commercial or financial relationships that could be construed as a potential conflict of interest.
